# Neuropsychological Rehabilitation for Traumatic Brain Injury: A Systematic Review

**DOI:** 10.3390/jcm14041287

**Published:** 2025-02-15

**Authors:** Carlos Ramos-Galarza, Jennifer Obregón

**Affiliations:** Facultad de Psicología, Pontificia Universidad Católica del Ecuador, Quito 170525, Ecuador; jnobregon@puce.edu.ec

**Keywords:** acquired brain injury, traumatic brain injury, neuropsychological treatment, cognitive rehabilitation

## Abstract

**Background/Objectives:** A traumatic brain injury (TBI) is a brain lesion caused by external or internal factors, resulting in cognitive, behavioral, physical, relational, and sensory sequelae, depending on the affected brain area and the severity of the injury. Within neuropsychological rehabilitation (NR), multiple methods have been developed that are aimed at restoring, compensating, and substituting deteriorated cognitive functions resulting from a TBI. This systematic review aimed to identify the state of the scientific literature regarding the efficacy of NR methods in individuals with a TBI. **Methods:** Articles were analyzed in the SCOPUS and PUBMED databases. Initially, 5347 studies were found. After applying inclusion and exclusion criteria, 17 articles remained and were included in the data extraction process. **Results:** Of the seventeen included articles, eleven employed randomized or semi-randomized controlled trials, five were clinical studies, and one was a comparative study, in which the percentage of computerized NR methods was 58.82% in the experimental and clinical groups. In contrast, traditional methods constituted 35.3%, and the remaining 5.88% conducted holistic NR. Ninety percent of the methods employed in these investigations showed efficacy. **Conclusions:** While most of the evaluated NR methods demonstrated efficacy, the analysis of these findings should not be isolated from variables such as the etiology and phase of the TBI, the intervention duration, and the symptoms treated. Furthermore, the NR implementation must be adapted to the specific context of each patient.

## 1. Introduction

A TBI refers to a brain lesion that disrupts the normal development of brain activity after a function has been acquired, which constitutes one of its most common etiologies [1,2]. It is caused by both exogenous and endogenous factors, resulting in cognitive, behavioral, physical, social, relational, and sensory sequelae, which depend on the affected brain area and the severity of the injury [3]. This type of injury often causes alterations in the cortical and subcortical regions of the brain [4], resulting in repercussions on various mental functions such as attention, memory, and language, which affect both the individuals experiencing them and their familial, social, and work environments [5].

The severity of a TBI is measured using the Glasgow Coma Scale parameters of eye-opening, verbal, and motor responses. It is categorized as mild (13–15 points), moderate (9–12 points), or severe (less than 9 points), and it plays a vital role in determining the prognosis and shaping treatment plans. For example, patients with a mild TBI do not require extensive neuropsychological rehabilitation, unlike those with a severe TBI, who often need a significant rehabilitation process that, in some cases, may extend for months or even years [6].

TBIs are a global public health concern, with a worldwide prevalence of around 64–74 million cases annually [7]. The incidence of TBIs is higher in the Latin American Caribbean region than anywhere else in the world [8], with an estimated prevalence rate of around 706 per 100,000 people [9]. In Ecuador, from 2004 to 2016, the rate of hospital admissions for TBIs stood at 70.68 per 100,000 individuals of all ages, with a higher incidence observed among men [10,11].

The global economic burden of TBIs is alarming, underscoring the critical need for the development of appropriate rehabilitation tools to facilitate, as much as possible, the reintegration of individuals with a TBI into productive activities. For instance, in TBI diagnoses and treatments worldwide, significant financial resources are allocated: approximately USD 4.2 trillion in the United States, EUR 84 billion in Spain, EUR 3.0 billion in Ireland, and CAD 120.7 million in Canada, among the most notable figures. Addressing TBIs is essential due to their substantial economic impact and their severe consequences in the personal, familial, and social context of the individuals affected by a TBI [12,13,14,15].

At a social level, patients with a TBI experience negative changes in their interpersonal relationships. They often struggle to maintain friendships or romantic relationships due to cognitive, behavioral, or emotional impairments. At a familial level, the economic and emotional burden resulting from the neurological sequelae of a TBI places significant stress on the patient’s relatives. In the workplace, individuals with a TBI face a substantial decline in their productive capacity, which may lead to reduced income or job loss. Within this framework, continued research on the diagnosis and rehabilitation of TBIs is essential to support those affected by this condition [16,17,18].

NR has been established as an intervention process for individuals experiencing cognitive or behavioral changes following a TBI, with the world wars playing a historically significant role in the development of such interventions. Following the Second World War, a growing interest emerged in establishing NR centers for military patients, marking one of the earliest milestones in recognizing this field of study. Germany, Russia, the United Kingdom, and the United States made significant contributions to this area. Subsequently, techniques for brain injury rehabilitation have evolved, transitioning from traditional paper-and-pencil-based methods to the contemporary use of cutting-edge technologies, such as virtual reality, specialized software, computerized tasks, and transcranial stimulation, among other innovative processes that continue to revolutionize the treatment of patients with a traumatic brain injury [19,20,21].

NR encompasses a set of interventions designed to restore cognitive, psychosocial, and emotional functions, including psychotherapy, psychoeducation, and strategies for reintegration into daily activities to the greatest extent possible [22]. NR comprises three main methods: restoration, compensation, and substitution. Restoration, also known as stimulation or retraining, involves a set of interventions that, through the direct training of the affected mental function, seeks to restore mental functionality based on the “bottom-up” mechanism [23].

Compensation refers to techniques that utilize preserved abilities and various mechanisms to enhance the impaired function. Its theoretical basis lies in the “top-down” mechanism, where intact brain areas compensate for the functions of the affected regions [24]. Regarding substitution, the use of external support strategies is aimed at compensating for the deficiency in the altered area. It employs devices or materials such as alarms, planners, lists, and calendars to assist the patient in organizing information and executing previously planned tasks [25]. Additionally, a program that has gained relevance in recent years is holistic NR proposed by Ben-Yishay and Diller, which emphasizes the role of the family in the treatment. It focuses on the creation of a therapeutic environment, a shared understanding of the patient’s needs and abilities to facilitate a coherent development of their identity, and the evaluation of the achieved outcomes [26].

Within the realm of NR, one study that stands out is the review conducted by Cicerone et al., which investigated neuropsychological and cognitive rehabilitation treatments in patients with a TBI. Through a systematic review incorporating class I evidence, the study demonstrated the efficacy of these interventions for rehabilitating functions such as perception, memory, language, and attention [27]. In this context, multiple methods have been developed to restore, compensate, and replace the cognitive functions impaired by a TBI. Traditional methods involve paper-and-pencil tasks, such as arithmetic calculations and reading aloud. Nonetheless, one limitation is their lack of ecological validity, since they may not accurately reflect real-world activities [28,29]. In contrast, innovative methods such as music therapy, telerehabilitation, computerized cognitive training, and virtual reality [4] have been developed.

However, varying results have been found regarding the efficacy of NR methods. For instance, the systematic review by Mateo-Fernández et al. [30] found that implementing SenseCam technology as a memory rehabilitation method in people with a TBI showed numerous improvements compared to alternative methods. Similarly, evidence has been found on the favorable effects of mindfulness-based interventions in the context of NR [31]. Furthermore, according to the review by Calderón-Chagualá et al. [32], it was revealed that both traditional tools and virtual reality are valid and reliable within NR methods. On the other hand, some methods lack sufficient scientific support regarding their efficacy in NR for individuals with a TBI, such as neurofeedback [33] and goal management training [34]. Regarding computerized cognitive training programs, although they offer a novel and promising method for addressing cognitive impairment, the efficacy of these programs in ameliorating such conditions in individuals with a mild TBI remains uncertain [35].

Thus, the previously mentioned background highlights the relevance of conducting theoretical research aimed at quantitatively systematizing the various NR methods for individuals with a TBI based on their efficacy. In this manner, this review will be beneficial in highlighting the effectiveness of various NR methods and their influence on improving the neuropsychological deficits of patients with a TBI, providing an updated overview of the interventions used in this population in the last years. Likewise, it will be handy for developing intervention plans that promote the appropriate treatment of neuropsychological symptoms in this population sector. The present systematic review contributes to the research on neuropsychological rehabilitation by providing clinical personnel working with this population with the necessary foundations to design intervention processes based on scientific evidence and effectiveness for treating TBIs.

## 2. Materials and Methods

**Figure 1:**
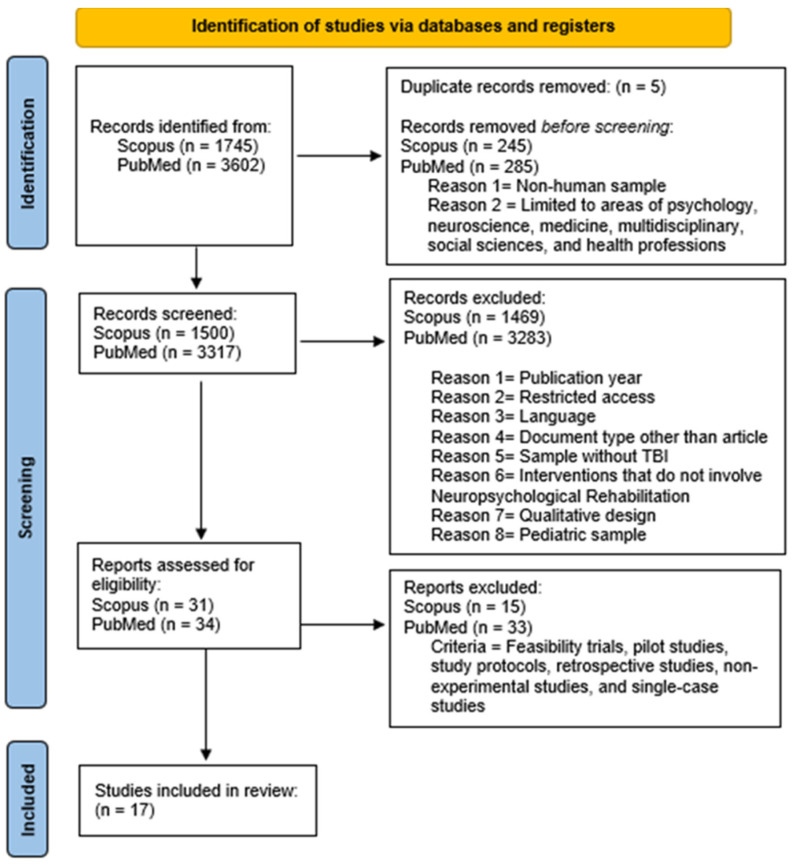
Flowchart of the conducted systematic review.

This research employed a systematic review design based on the PRISMA standards. The sections of the methodology applied in the review process are described below (Figure 1).

### 2.1. Identification (Including Duplication)

The databases used for this procedure were SCOPUS and PUBMED, using the following keywords: (“Neuropsychological” AND “Rehabilitation”) AND (“Traumatic” AND “Brain” AND “Injury”) OR (“Acquired” AND “Brain” AND “Injury”). At first, a total of N = 5347 articles were obtained. Afterward, duplicate articles (N = 5) were identified and excluded, and N = 530 were left out after applying the following search filters: human sample and subject related to medicine, psychology, neuroscience, health professions, social sciences, or multidisciplinary areas.

### 2.2. Screening and Eligibility

In this stage, the remaining studies were analyzed according to the inclusion criteria (the use of NR methods, the Spanish or English language, a publication year between 2019 and 2024, an adult sample with a TBI, and a quantitative design) and exclusion criteria (other languages, publication outside the period of 2019–2024, conditions other than a TBI, a pediatric sample, paid access, systematic reviews, meta-analyses, books, grey literature, and a qualitative design), resulting in the exclusion of N = 4752 articles.

### 2.3. Included Studies and Procedure

This study commenced with the formulation of the research questions. Subsequently, a comprehensive review process was conducted across relevant databases. Following this, an extraction table was developed, and the data were systematically analyzed. In this phase, N = 17 [35,36,37,38,39,40,41,42,43,44,45,46,47,48,49,50,51] articles were included in this research. The statistical analyses performed on the data included calculating central tendency and dispersion measures. The process concluded with a detailed discussion of the findings. Lastly, it is essential to note that this systematic review was formally registered on the Open Science Framework platform (https://doi.org/10.17605/OSF.IO/27VHM (accessed on 20 January 2025)).

### 2.4. Bias Analysis

One reviewer supervised the research process, accepting or rejecting the information found in the articles from an extraction table, which included categories such as the research design, the year and country, the sample size used, the type of NR method, and the intervention time, among others. These data are gathered in Appendix A.

## 3. Results

The following section contains statistical graphs that allow for an analysis of the variables related to the research objectives concerning the NR methods in the included scientific articles. The methodological and demographic characteristics of the studies were evaluated, and the instruments and symptoms addressed in the analyzed studies were also examined. Additionally, measures such as the mean were calculated and considered to better understand the obtained data regarding aspects such as the central tendency.

### 3.1. ABI Etiology

**Figure 2:**
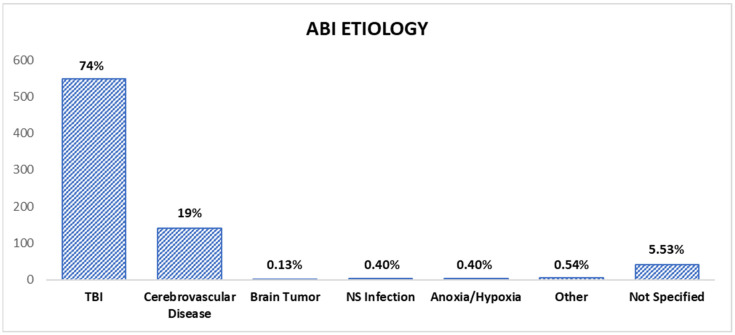
Types of ABI etiology.

Within the total sample of 742 individuals in the analyzed studies, the most predominant etiology was a traumatic brain injury in 549 patients, followed by cerebrovascular disease in 141. The remaining causes included a nervous system infection (3), anoxia or hypoxia (3), and a brain tumor (1), as indicated in Figure 2.

### 3.2. Type of NR Method Employed

**Figure 3:**
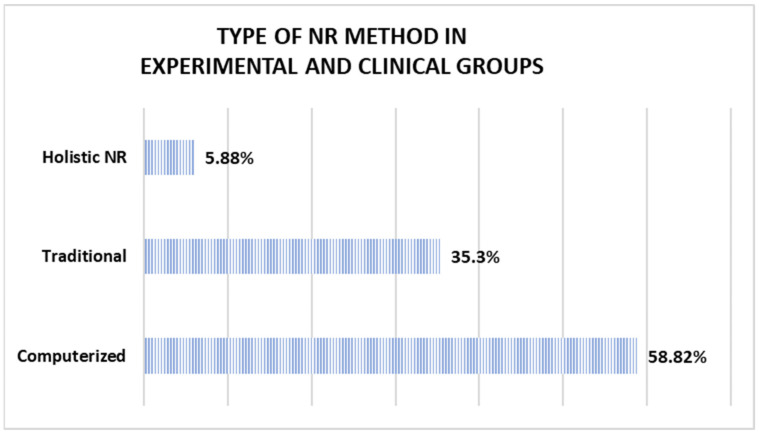
NR methods in experimental groups and clinical groups.

**Figure 4:**
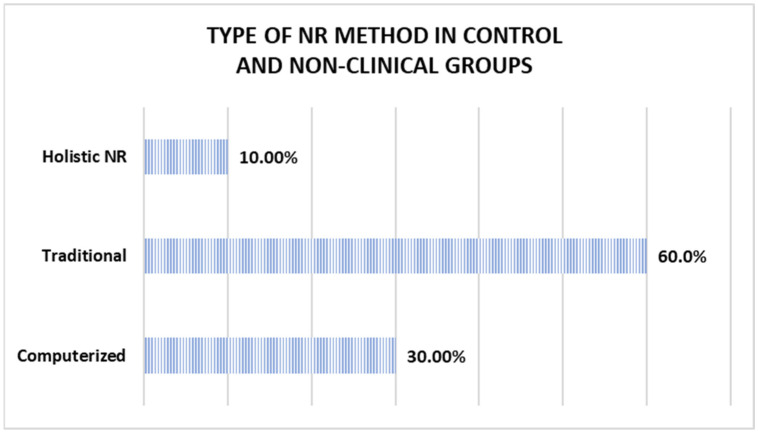
NR methods in control and non-clinical groups.

Out of the 17 methods employed in the experimental groups and the clinical study, 6 used traditional interventions, 10 implemented computerized programs, and 1 implemented a holistic NR program, as shown in Figure 3. In contrast, of the ten methods used in the control and non-clinical groups, six were traditional, three were computerized, and one used holistic NR (as illustrated in Figure 4).

### 3.3. ABI Phase from the Participants

**Figure 5:**
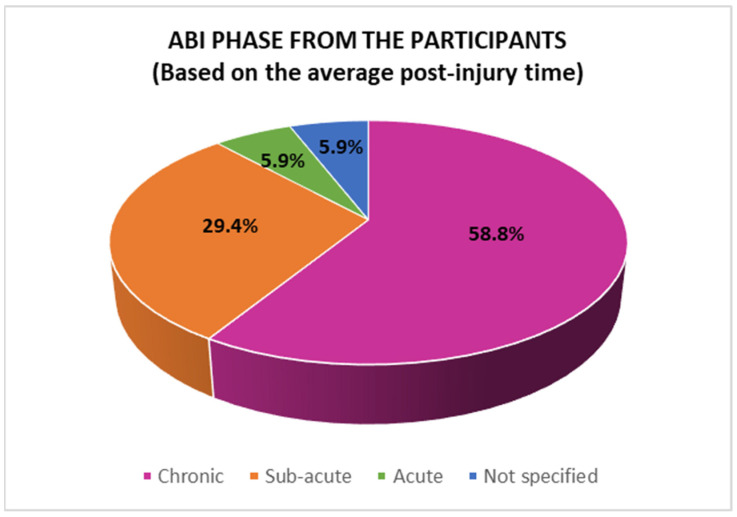
ABI phase from the sample.

The average number of days post-injury was used to define the ABI’s acute, sub-acute, and chronic phases. Ten studies used methods to intervene in the chronic phase, five in the sub-acute phase, and one in the acute phase, while one study did not specify these data, as shown in Figure 5.

### 3.4. Intervals of the Number of Sessions

**Figure 6:**
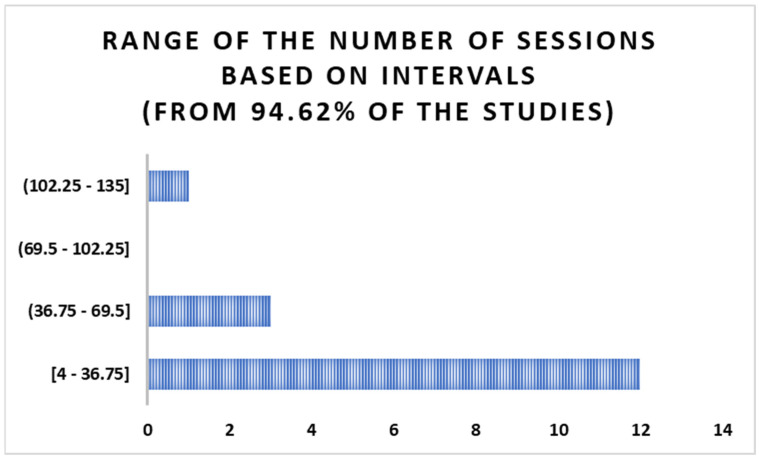
Intervals of the number of sessions conducted by the NR methods.

The range of the number of sessions was calculated in intervals for 94.62% of the studies; as in the research by Terneusen et al. [39], this number was only expressed as a range between 1 and 162 sessions. As depicted in Figure 6, in the 17 studies examined, the number of sessions most frequently fell within the range of 4 to 36.75 in 12 studies, followed by the interval of 36.75 to 69.5 in 3 studies and 102.25 to 135 in 1 study.

### 3.5. Treated Conditions

**Figure 7:**
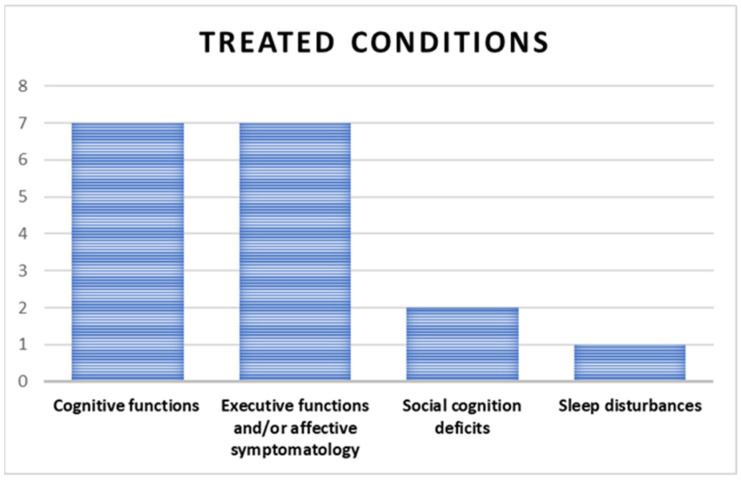
Conditions treated by the NR methods.

The most frequently treated conditions were cognitive functions (seven studies), executive functions, and affective symptomatology (seven studies), followed by social cognition deficits (two studies). Lastly, only one study addressed sleep disturbances, as demonstrated in Figure 7.

### 3.6. Assessment Tool Categories

**Figure 8:**
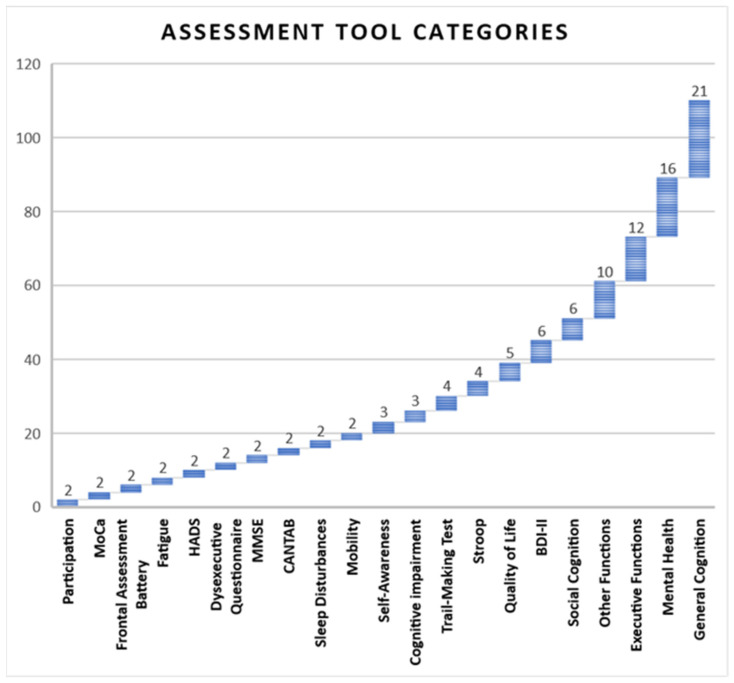
Classification of assessment tools.

As illustrated in Figure 8, out of the 110 assessment tools, the most frequently measured category was general cognition, followed by mental health, executive functions, and tests that assessed other types of neuropsychological functions.

### 3.7. NR Strategies

**Figure 9:**
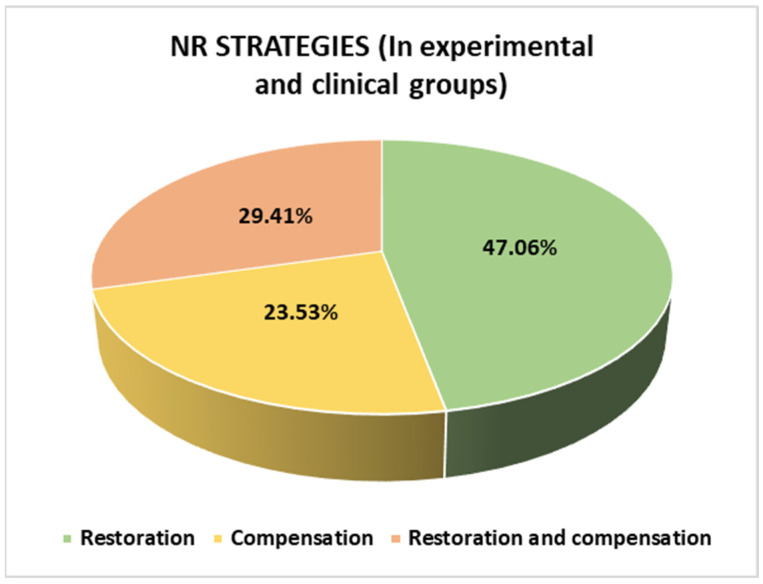
NR strategies from methods in experimental and clinical groups.

In the experimental and clinical studies, eight employed restoration strategies, four used compensation strategies, and the remaining five utilized both types, as shown in Figure 9.

### 3.8. Efficacy of the Methods

**Figure 10:**
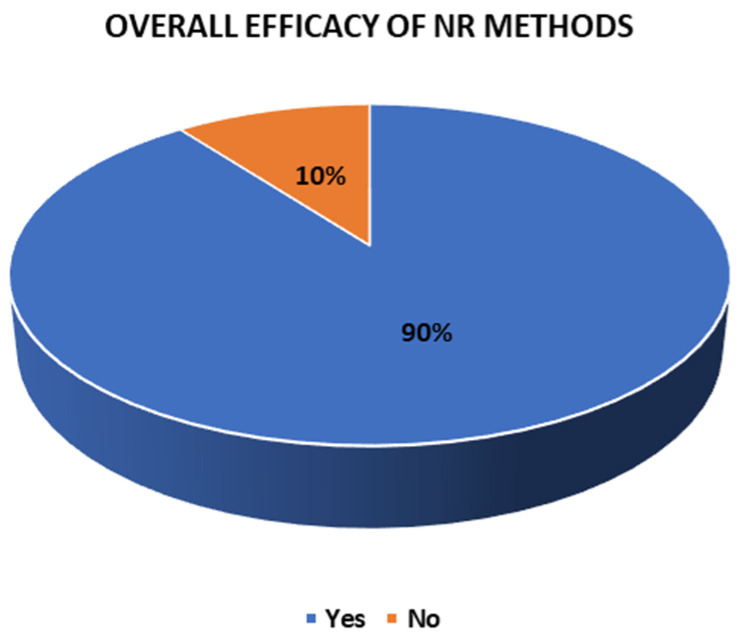
Overall efficacy of the NR methods.

**Figure 11:**
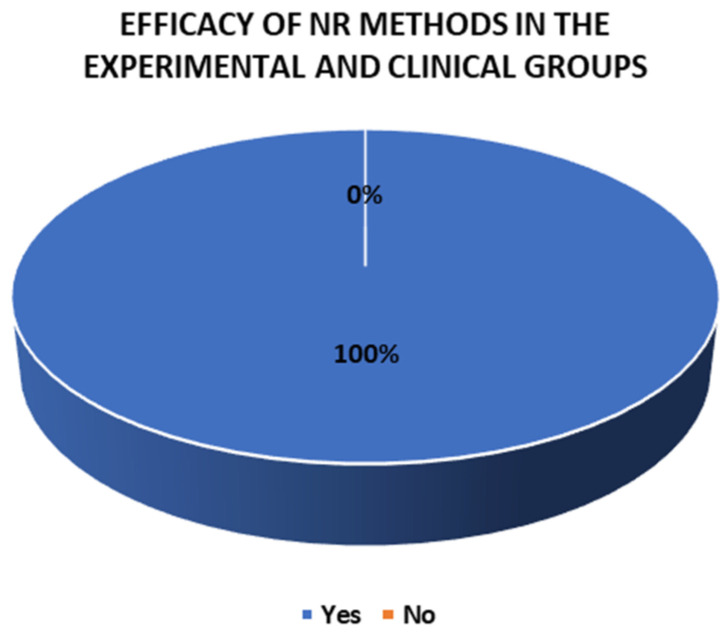
Efficacy of the NR methods in the experimental and clinical groups.

**Figure 12:**
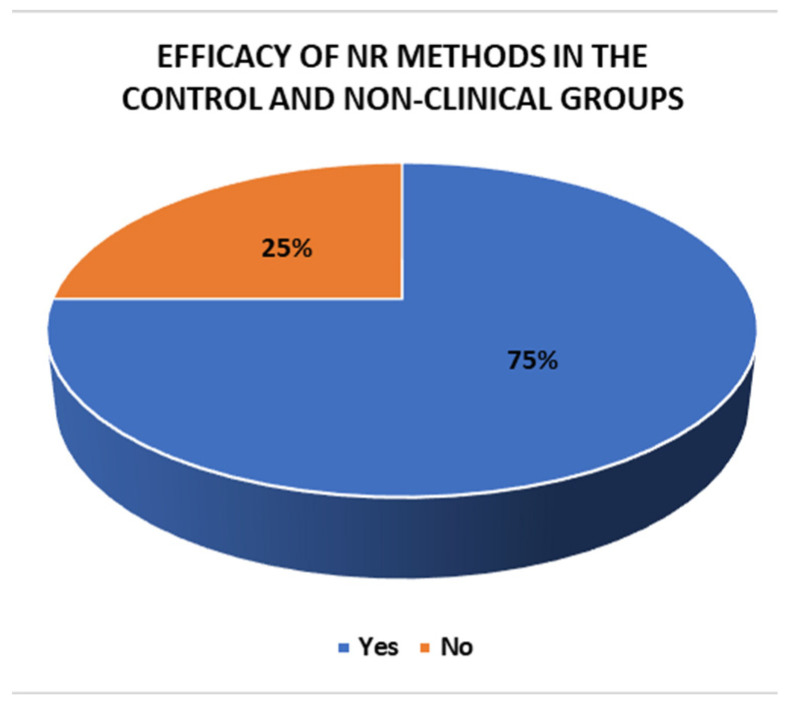
Efficacy of the NR methods in the control and non-clinical groups.

Of the 29 methods employed in the experimental and clinical groups and the control and non-clinical groups, 26 showed efficacy in their results and 3 did not. Specifically, all the NR methods (17) in the experimental and clinical groups demonstrated their effectiveness. In contrast, in the control and non-clinical groups, nine methods showed efficacy and three did not: Rehacom [40], psychoeducation only [41], and treatment as usual [51]. These findings are explored in Figure 10, Figure 11 and Figure 12.

## 4. Discussion

This study consisted of a quantitative systematic review with a descriptive scope. Based on the eligibility criteria, it aimed to characterize articles from the last five years demonstrating the efficacy of NR methods in individuals with a TBI. Thus, eleven of the included articles used an experimental or quasi-experimental design, while five were clinical and one was comparative. These studies found that 90% of the interventions performed resulted in an improvement in the TBI conditions. However, these findings cannot be considered generalizable due to the heterogeneity in the characteristics of the samples of the included studies, and the complexity this necessitates a standardized comparison between methods. This is reflected in the diversity of variables, such as the etiology of TBIs, the number of post-injury days, the duration of the intervention, and the symptoms treated.

In this regard, it is worth emphasizing that, to date, the specific factors and therapies (or their combinations) that are most effective in NR are not entirely clear [52]. This is related to factors such as the limited conceptual delineation of many therapies [53] and the high number of studies with a low methodological quality when evaluating the efficacy of specific methods [54]. For this reason, the present research underscores the importance of describing the characteristics in producing scientific literature on this topic to contribute to a better understanding of the factors that influence efficacy.

Regarding the methodological characteristics of the present review, out of 17 included studies, 11 were randomized controlled or semi-randomized trials, which, according to Wilson et al. [4], constitute the preferred design types for the scientific evidence of NR methods due to their quality parameters. In terms of the geographical location of the research, 47.06% was conducted in Europe, while 23.53% was in North America, 23.53% was in Asia, and the remaining 5.88% was in Brazil, with these studies being published between 2019 and 2024. Additionally, regarding the sample type, cases of patients with a TBI (73.99%) or a stroke (19.00%) stand out.

For the intervention times from the acquisition of a TBI, it was found that, on average, 58.82% of the studies performed the treatment in the chronic phase of the ABI, while 29.41% did so in the post-acute phase and 5.88% did so in the acute phase. In this regard, Ibáñez et al. [5] suggest that it is in the post-acute phase (six or more weeks after an ABI) when NR occurs per se. The treatment, which is usually based on restoration, should be comprehensive and complete. In contrast, given that the sequelae persist for a year or more after the injury, the chronic phase is oriented towards implementing compensatory and substitution strategies.

On the other hand, the instruments primarily assessed the general cognition (19.09%) and mental health (14.55%), highlighting their adequate psychometric properties. According to Muñoz Marrón et al. [11], this aspect helps to increase the efficacy of the evaluations and the representativeness of the findings. Respecting the number of sessions, these ranged from 1 to 162, with the most frequent being between 4 and 36.75 sessions.

Regarding the type of intervention methods, in the experimental groups, 35.3% were traditional. Meanwhile, 58.82% were computerized types and 5.88% applied holistic NR. As a point of comparison, Spreij et al. [55] and Bogdanova et al. [56] found favorable changes from using computerized programs in working memory, attention, and executive functions, respectively. In contrast, the research by Laver et al. [57] and Chen et al. [58] did not find conclusive results on the efficacy of telerehabilitation in stroke patients. Thus, although this study reflected the efficacy of 90% of the NR methods included, these results should be analyzed while considering the various factors and parameters that influence the recovery from a TBI, such as the magnitude and severity of the injury, the premorbid characteristics, and possible comorbidities [59].

One of the challenges arising from the present research is the development of clinical care services for patients with a traumatic brain injury (TBI). For instance, the study conducted by Dasic et al. [60] highlights the need for neurotrauma services to ensure an effective intervention process for individuals with a TBI. This aspect of research demands urgent attention, as TBIs are the leading cause of brain damage worldwide. In contexts such as Latin America [61], neuropsychological rehabilitation services are still in development, making it essential to continue generating scientific evidence in this field of study.

The limitations of the current systematic review must be taken into account. Firstly, case studies were excluded from the included articles for representativeness motives. Additionally, articles with restricted access requiring payment were not considered. Similarly, the geographical regions of the included articles were limited to Europe, Asia, and North America, and studies with qualitative methodologies were not included. Furthermore, the heterogeneity of the NR methods and the variability in the conditions under which patients received interventions made it difficult to standardize and make deeper comparisons. In this sense, future research could conduct systematic reviews on the efficacy of NR methods within case study frameworks and include articles with qualitative or mixed-method designs. Likewise, they could consider applying the various NR methods in regions such as South America and Africa, adapting them to the contextual conditions, and considering the resources available compared to industrialized countries. Finally, future research interests include developing a neuropsychological rehabilitation procedure specifically designed for patients with a TBI. This procedure will incorporate the key findings of this research to create an effective protocol supported by prior empirical evidence that can benefit individuals with a TBI.

## 5. Conclusions

The findings of this systematic review highlight the efficacy of NR methods in addressing cognitive deficits in TBIs, with the evaluated methods showing positive outcomes. However, the effectiveness of these interventions is closely tied to factors such as the etiology and phase of the TBI, the framework of the intervention, and the specific symptoms being treated. While computerized NR methods were predominant in treating people with TBIs, traditional and combined approaches also demonstrated significant benefits. These results underscore the importance of tailoring NR strategies to patients’ individual needs and contexts, ensuring that rehabilitation programs are flexible and patient-centered. Future research should continue to explore the interplay between these variables to optimize the rehabilitation outcomes for individuals with a TBI.

## Data Availability

The raw data supporting the conclusions of this article will be made available by the authors without undue reservation.

## Appendix A

**Table A1:** Information extraction table.

No.	Title	Authors	Research Design	Year	Country	ABI Etiology	Sample Size	Average Age	Average Post-Injury Time	NR Method	NR Strategies	Number of Sessions	Treated Symptoms	Assessment Tools	Results
1	Efficacy of computerized vs. Traditional cognitive interventions for the treatment of chronic mTBI symptoms among service members	Darr et al. [35]	Comparative Study	2024	USA	TBI	65	32.85 years	1460 days	Group 1: computerized cognitive training (CCT) program. Group 2: clinician-run, manualized cognitive rehabilitation.	CCT: restoration and compensation	12	Cognitive functioning; perception of neurobehavioral difficulties.	1. KBCI 2. PASAT 3. SDMT	CCT can be used as an effective method to treat chronic cognitive complaints in mTBI. However, it is likely most beneficial when incorporated into existing, evidence-based methods of CR.
2	Virtual reality–based music attention training for acquired brain injury: A randomized crossover study	Jeong et al. [36]	Randomized Crossover Trial	2024	South Korea	Stroke: 16 TBI: 6 Hypoxia: 1	23	46.9 years	1638 days	Group 1: virtual reality-based music attention training (VR-MAT), followed by conventional cognitive training (CCT). Group 2: CCT followed by VR-MAT.	VR-MAT: restoration	20	Cognitive impairment.	1. TMT 2. CANTAB 3. CDR 4. GDS 5. MMSE	VR-MAT demonstrated efficacy as an intervention and assessment tool for cognitive rehabilitation in patients with an ABI.
3	Neuropsychological correlates of PTSD and depressive symptom Improvement in compensatory cognitive training for veterans with a history of mild traumatic brain injury	Clark et al. [37]	Clinical Study	2024	USA	TBI	37	36.9 years	Not specified	1. Experimental group: compensatory cognitive training (CCT).	Compensation	10	PTSD and depression.	1. PCL-M 2. BDI-II 3. D-KEFS 4. HVLT-R 5. WAIS-IV 6. WRAT-4	Cognitive training may bolster skills that are helpful for PTSD and depressive symptom reduction.
4	Speed of processing training to improve cognition in moderate to severe TBI: a randomized clinical trial.	Chiaravalloti et al. [38]	Randomized Clinical Trial	2024	Italy	TBI	46	41.45 years	3942 days	1. Experimental group: speed of processing training (SOPT). 2. Control group: placebo.	SOPT: restoration	10	Processing speed.	1. UFOV 2. SDMT 3. WAIS-IV 4. CVLT II	SOPT improved task performance on a task similar to the training task (UFOV), but benefits did not extend to improvement in neuropsychological tests of processing speed.
5	Socratic guided feedback therapy after acquired brain injury: A multicenter randomized controlled trial to evaluate effects on self-awareness	Terneusen et al. [39]	Multicenter Randomized Controlled Trial	2024	Netherlands	Stroke: 34 TBI: 22 Anoxia/Hypoxia: 2 NS Infection: 2 >2 of the Above: 2 Not Specified: 2	64	50.8 years	82 days	1. Experimental group: Socratic guided feedback therapy. 2. Control group: treatment as usual.	Socratic guided feed-back therapy: compensation	Between 1 and 162	Self-awareness.	1. SRSI 2. PCRS 3. MOT-Q 4. PRPS 5. HADS 6. SSQOL-12 7. USER-P	Both groups improved in terms of self-awareness over time.
6	Treating social cognition impairment with the online therapy ’SoCoBo’: A randomized controlled trial including traumatic brain injury patients.	Lohaus et al. [40]	Randomized Controlled Trial	2024	Germany	TBI	43	44.9 years	2529 days	1. Experimental group: SoCoBo. 2. Control group: RehaCom.	SoCoBo: compensation	48	Social cognition deficits.	1. ERI 2. IRI 3. GERT 4. HPP-S 5. ISK-K 6. TAS-20 7. Social Cognition Test Battery 8. RWT 9. Wechsler Memory Scale: Digit Span 10. AVLT 11. Stroop 12.DESC 13. FLZ 14. SIAS 15. STAI	The SoCoBo group, but not the RehaCom group, showed significant improvements in facial emotion recognition and self-rated empathy, which were associated with increased life satisfaction. ToM and social problem-solving did not improve. Additionally, general cognition did not improve in either group.
7	Heart rate variability biofeedback for mild traumatic brain injury: A Randomized- controlled study	Lu et al. [41]	Randomized Controlled Trial	2023	Taiwan	TBI	41	37.2 years	3.3 days	1. Experimental group: heart rate variability biofeedback (HRVB). 2. Control group: psychoeducation.	HRVB: restoration	10	Executive functioning, information processing, verbal memory, affective neuropsychological functioning, and heart rate variability.	1. FAB 2. SVF 3. WSLT 4. PASAT 5. TMT 6. Checklist of Post-Concussion Symptoms (CPCS) 7. DEX 8. Beck Anxiety Inventory 9. BDI-II 10. National Taiwan University Irritability Scale (NTUIS)	The experimental group evidenced improvements in executive functioning, information processing, verbal memory, neuropsychological emotional functioning, and heart rate variability (HRV), while the psychoeducation group showed no change.
8	Benefits of telerehabilitation for patients with severe acquired brain injury: Promising results from a multicenter randomized controlled trial using nonimmersive virtual reality	Calabrò et al. [42]	Multicenter Randomized Controlled Trial	2023	Italy	Stroke: 28 TBI: 12	40	48.12 years	347.66 days	1. Experimental group: telerehabilitation (VRRS HomeKit device). 2. Control group: usual territorial rehabilitation treatment.	VRRS HomeKit: restoration	60	Functional alterations at the motor level, frontal/executive capacities, visuospatial memory, verbal fluency, reasoning, and anxiety and depression symptoms.	1. Barthel Index (BI) 2. Tinetti Scale (TS) 3. Modified Ashworth Scale (MAS) 4. MoCa 5. Frontal Assessment Battery (FAB) 6. BDI-II 7. SF-36 8. PGWBI 9. Caregiver Burden Inventory (CBI)	Both the VRRS and control groups improved in global functional, cognitive, and general health. However, only the VRRS group improved in motor and executive functions, with a significant reduction in anxiety and depression symptoms.
9	Holistic neuropsychological rehabilitation: Cognitive evolution and quality of life of patients with acquired brain injury.	Gómez Pulido [43]	Clinical Study	2023	Spain	TBI: 9 Stroke: 11	20	59.75 years	494.58 days	Holistic NR.	Restoration, compensation, and substitution	135	Attention, memory, and executive functions; specific social skills.	1. WAIS-IV 2. WMS-III 3. ToL 4. Wisconsin Card Sorting Test (WCST) 5. WHOQOL-BRiEF	Improvements were evidenced in the performance of attention, working memory, executive functions, and quality of life.
10	Cerebrolysin and repetitive transcranial magnetic stimulation (rTMS) in patients with traumatic brain injury: A three-arm randomized trial	Verisezan Rosu et al. [44]	Three-Arm Randomized Controlled Trial	2023	Romania	TBI	93	52.12 years	Within 30 days	Groups: 1. Cerebrolysin (CRB) and transcranial magnetic stimulation (rTMS). 2. Cerebrolysin (CRB) and sham rTMS (SHM). 3. Placebo and sham rTMS (SHM).	Restoration	10	Cognitive and functional symptoms.	1. Stroop Color-Word Test 2. MoCA 3. WAIS 4. TMT 5. CANTAB 6. Hamilton Anxiety Rating Scale (HARS) 7. Hamilton Rating Scale for Depression (HDRS)	The combined intervention of rTMS and Cerebrolysin was safe and well tolerated by patients with a TBI.
11	The effectiveness of computer-assisted cognitive rehabilitation and the degree of recovery in patients with traumatic brain injury and stroke	Jung et al. [45]	Clinical Study	2021	South Korea	TBI: 30 Stroke: 32	62	58.41 years	67.58 days	Computer-assisted cognitive rehabilitation (Comcog).	Restoration and compensation	30	Cognitive impairment.	1. Computerized Neuropsychological Test (CNT) 2. MMSE 3. Modified Barthel index (MBI)	Patients with a TBI or a stroke showed significant changes in cognitive functions. The stroke group showed a high difference value.
12	The impact of multimodal cognitive rehabilitation on executive functions in older adults with traumatic brain injury	Cisneros et al. [46]	Semi-Randomized Controlled Trial	2021	Canada	TBI	37	64.5 years	695.5 days	1. Experimental group: cognitive enrichment program (CEP). 2. Control group: holistic NR.	CEP: Restoration, compensation, and substitution	24	Executive functions and reintegration to daily life activities.	1. Six Elements Task-Adapted [SET-A] 2. D-KEFS Sorting Test 3. Stroop 4. Dysexecutive Questionnaire (DEX)	Improvements in executive functioning were evidenced in the experimental group, with a positive impact on daily activities.
13	A randomized clinical trial of plasticity based cognitive training in mild traumatic brain injury	Mahncke et al. [47]	Randomized Clinical Trial	2021	USA	TBI	83	33.8 years	2264.5 days	1. Experimental group: Self-administered computerized cognitive training program based on plasticity. 2. Control group: computerized games.	Self-administered computerized cognitive training program based on plasticity: restoration	65	Cognitive functions; attention.	1. ANAM TBI Battery Score 2. RNBI 3. PTSD Checklist C (PCL-C) 4. BDI-II 5. FrSBe 6. CFQ 7. NSI 8. MPAI	Statistically equivalent improvements were observed in both groups in depressive and cognitive symptoms.
14	Cognitive retraining in traumatic brain injury: Experience from tertiary care center in southern India	Afsar et al. [48]	Prospective Clinical Study	2021	India	TBI	12	32.33 years	345.84 days	Cognitive retraining (CR).	Restoration	20	Cognitive impairment.	1. NIMHANS 2. Perceived Stress Scale 3. Rivermead Post-Concussion Symptom Questionnaire 4. WHOQL-BRIEF 5. Visual Analogue Scale	CR can be helpful in improving cognition, symptom reporting, and quality of life in moderate to severe TBIs.
15	An integrative neuro-psychotherapy treatment to foster the adjustment in acquired brain injury -a randomized controlled study	Urech et al. [49]	Randomized Controlled Trial	2020	Switzerland	Stroke: 20 TBI: 3 Brain Tumor: 1 Encephalitis: 1	25	48.3 years	509.2 days	1. Experimental group: integrative neuro-psychotherapy treatment (Standard PLUS). 2. Control group: standard neuropsychological treatment.	Standard PLUS: restoration and compensation	20	Depressive symptoms from adjustment disorder, cognitive functions funcional deficits, and coping.	1. BDI-II 2. WHOQOL-BREF 3. ADS 4. Awareness Questionnaire (AQ) 5. Trier Illness Coping Scales (TSK) 6. ERSQ 7. RAS 8. Mental Fatigue Scale (MFS)	Both treatments were effective, although there was no significant difference between them.
16	Transcranial photobiomodulation therapy in the cognitive rehabilitation of patients with cranioencephalic trauma	Costa Carneiro et al. [50]	Multidisciplinary Clinical Study	2019	Brazil	TBI	10	37.8 years	From 4 months to 4 years	Transcranial photobiomodulation therapy.	Restoration	18	Cognitive function.	1. BDI-II 2.BAI 3. Stroop Test 4. TMT 5. Symbol Digit Test 6. RAVLT 7. Complex Rey Figure 8. F-A-S	The assessment results suggest some improvement of cognitive function in patients with a TBI.
17	A short add-on sleep intervention in the rehabilitation of individuals with acquired brain injury: A randomized controlled trial.	Pilon et al. [51]	Randomized Controlled Trial	2023	Netherlands	Not Specified	41	47.3 years	1727.3 days	1. Experimental group: brief complementary treatment of sleep disorders, based on cognitive–behavioral therapy for insomnia. (CBT-I) plus treatment as usual. 2. Control group: treatment as usual.	CBT-I: compensation	4	Sleep disturbances.	1. PSQI 2. DMFS 3. HADS 4. DBAS-16 Brief Version	The experimental group had improvements in sleep quality, manifested fewer dysfunctional beliefs and attitudes about sleep, and was better able to cope with fatigue compared to the control group.
